# A Dual-Mode Imaging Nanoparticle Probe Targeting PD-L1 for Triple-Negative Breast Cancer

**DOI:** 10.1155/2022/2431026

**Published:** 2022-05-31

**Authors:** Xiajin Li, Yinan Ji, Miao Chen, Siyi Zhang, Ziyu Wang, Danke Su, Ningbin Luo

**Affiliations:** ^1^Departments of Radiology, Guangxi Medical University Cancer Hospital, 71 Hedi Road, Nanning 530021, Guangxi Zhuang Autonomous Region, China; ^2^Department of Breast Surgery, Guangxi Medical University Cancer Hospital, 71 Hedi Road, Nanning 530021, Guangxi Zhuang Autonomous Region, China

## Abstract

Chemotherapy has remained the mainstay of treatment of triple-negative breast cancer; however, it is significantly limited by the associated side effects. PD-1/PD-L1 immune checkpoint inhibition therapy (ICI) has been a breakthrough for this patient population in recent years. PD-L1 expression is crucial in immunotherapy since it is a major predictor of PD-1/PD-L1 antibody response, emphasizing the significance of monitoring PD-L1 expression. Nonetheless, it is hard to assess the expression of PD-L1 before surgery, which has highlighted the urgency for a precise and noninvasive approach. Herein, we prepared a dual-mode imaging nanoparticle probe to detect PD-L1. The particle size, zeta potential, biocompatibility, and imaging ability of NPs were characterized. The synthesized NPs showed slight cytotoxicity and good T2 relaxivity. The targeted NPs accumulated more in 4T1 cells than nontargeted NPs *in vitro*. The *in vivo* experiment further demonstrated the distribution of targeted NPs in tumor tissues, with changes in NIRF and MR signals observed. Our study indicated that SPIO-aPD-L1-Cy5.5 NPs can be used to monitor PD-L1 expression in breast cancer as NIRF/MR contrast agents.

## 1. Introduction

Triple-negative breast cancer (TNBC) is a subtype of breast cancer negative for PR/HR/Her-2 receptors, accounting for 10–20% of breast cancers. It has been established that TNBC exhibits a highly aggressive phenotype accounting for its poor prognosis [1–3]. PD-1/PD-L1 immune checkpoint inhibition therapy (ICI) is one of the current hot topics in research on TNBC treatment. Studies have shown that PD-L1 expression is a predictor of pathologic complete response (pCR) for neoadjuvant chemotherapy in breast cancer patients [4, 5], and effective response rates correlate with the immune checkpoint inhibitor treatment [6]. Accordingly, monitoring the PD-L1 expressions on the surface of tumor cells has huge prospects for optimizing the current patient management and treatment. However, the clinical translation of this approach is subject to many limitations, including the invasive nature of the approach for specimen acquisition and the time limit associated with the results. Besides, the results varied with differences in the detection method and time [7, 8].

Molecular imaging enables noninvasive monitoring of changes in potential molecular targets of tumor cells. In recent years, contrast agents have been developed to detect tumors and improve treatment efficacy, involving recognizing specific receptors on the surface of tumor cells, such as *α*_v_*β*_3_ [9, 10] and EGFR [11, 12], which have yielded excellent results. It has been established that near-infrared fluorescence imaging (NIRF) has excellent sensitivity, specificity, and safety profile. Nonetheless, it is limited by the penetration depth of the laser [13, 14]. In contrast, MRI can provide high spatial resolution with outstanding contrast features in soft tissues but is limited by its high false-positive rate [15, 16]. Interestingly, combining these techniques exhibited a synergistic effect that has been utilized for the detection of tumors [17, 18] and atherosclerosis [19, 20].

Superparamagnetic iron oxide (SPIO) nanoparticles are among the highest-ranked nanoparticles in medicine due to their unique physicochemical property (superparamagnetism) as well as their established biocompatibility and stability in aqueous solutions [21, 22]. Moreover, it has been intensively studied to develop contrast agents in MRI [23, 24]. In comparison to molecular probes with Gd^3+^ as the signal unit, SPIO has some upsides. (1) The signal intensity of SPIO is higher than Gd chelate contrast agents at the same concentration. (2) SPIO absorption by the body is accompanied by efficient biodegradation and iron homeostasis mechanisms for treating the free irons [25], and that avoids nephrogenic systemic fibrosis (NSF) resulting from gadolinium exposure [26]. Therefore, we synthesized a dual-mode contrast agent, SPIO-aPD-L1-Cy5.5, which used SPIO as a core for MR and then conjugated Cy5.5 with NIRF. Besides, we further investigated the stability, toxicity, and targeting accuracy of SPIO-aPD-L1-Cy5.5 and evaluated the predictive efficacy of SPIO-aPD-L1-Cy5.5 for PD-L1 expression of TNBC.

## 2. Materials and Methods

### 2.1. Synthesis of SPIO-aPD-L1-Cy5.5 Nanoparticles

Free amino groups of antibody IgG and anti-PD-L1 can react with the active group of PEG-COOH-coated Fe_3_O_4_ and NHS-Cy5.5 to form stable amide linkages. Take 20 mg solution of PEG-COOH-coated Fe_3_O_4_ (Nanoeast, China), replace the buffer with 0.02 M MES solution, and adjust the pH to 5.5. Then, 2 mg PD-L1 antibody (clone 10F.9G2) or IgG antibody (for the control group) was added, and the mixture was incubated at 26°C for 30 min in a shaker. Subsequently, 4 mg EDC was added to the mixture and incubated overnight in a shaker at 26°C. The solution was removed, and a magnetic separation column was used to remove the free antibody and rinse out the stagnant magnetic beads. 1 ml of the solution was treated at a time, and the obtained colorless solution was then measured. NHS-Cy5.5 was dissolved in anhydrous DMF and diluted to a 20 mg/mL solution. The magnetic beads were taken after coupling the PD-L1 antibody, and the volume was made to 10 mL. The NHS-Cy5.5 solution was added to the mixture and incubated at 26°C for 5 h in a shaker. The free Cy5.5 was removed using a 100 K ultrafiltration tube (5000 rpm, 10 min) until a clear colorless solution was observed. The volume of the product was made to 20 mL at a concentration of 1 mg/ml.

### 2.2. Nanoparticle Characterization

The nanostructure of the nanoparticles was determined by a transmission electron microscope (TEM; FEI, USA). The zeta potential and size of the nanoparticles were studied by dynamic light scattering using a Zetasizer Nano ZS (Malvern Instruments, UK). The emission and excitation spectra were recorded by a fluorescence spectrometer (Alpha II, Bruker, Switzerland).

### 2.3. Cellular Toxicity Study *In Vitro*

The CCK-8 method was used to examine the cytotoxicity of the molecular probe on 4T1 cells. The 4T1 cells were seeded in a 96-well plate at a 5 × 10^3^ cells/well density and cultured overnight. The adherent cells were then incubated with 100 *μ*L of the corresponding medium containing SPIO-aPD-L1-Cy5.5 of various Fe concentrations (0, 5, 10, 20, 50, and 100 *μ*g/ml) for 24 h. Subsequently, the cells were washed three times with PBS, and then, 90 *μ*L of fresh medium and 10 *μ*L of CCK8 reagent were added. The 96-well plates were then incubated at a constant temperature for 2 h. In addition, a blank group (only add the 90 *μ*L of fresh medium and 10 *μ*L of CCK8 reagent) was set up. A microplate reader was used to record the absorbance at a wavelength of 450 nm. The cell viability was calculated using the following formula:

(1) $$\begin{array}{c} \text{Cell viability}=\left[\frac{\left(A_{e}-A_{b}\right)}{\left(A_{c}-A_{b}\right)}\right]\times 100\%, \end{array}$$

where *A*_e_ is the absorbance of the experimental well, *A*_c_ is the absorbance of the control well, and *A*_b_ is the absorbance of the blank well. The experiment was repeated three times.

### 2.4. Cellular Uptake Study *In Vitro*

To evaluate the cellular uptake of the nanoparticles, 4T1 cells were seeded onto 35 mm glass-bottom culture dishes (MatTek, USA) at a density of 1 × 10^5^ cells/mL overnight. Then, the cells were incubated with SPIO-aPD-L1-Cy5.5 NPs (5 *μ*g/ml, 1 mL) at pH 7.4 for 4 h, and SPIO-IgG-Cy5.5 NPs were used as controls. To further validate the active targeting of the nanoparticles, a blocking group was made, which was designed to add antibody PD-L1 (Bioss, China) before adding SPIO-aPD-L1-Cy5.5. After the incubation, the cells were then washed three times in PBS, and DAPI was used to stain the nuclei for 10 minutes. Fluorescence images were acquired by confocal laser scanning microscopy (TCS SP8, Leica, Germany).

### 2.5. *In Vivo* and *In Vitro* MRI and NIRF and Prussian Blue Iron Staining

All experiments followed animal ethics of the guiding opinions on the treatment of laboratory animals issued. All female Balb/c nude mice (aged 4 weeks) were obtained from the Experimental Animal Center of Guangxi Medical University. 4T1 cells were injected subcutaneously in the right flank at a concentration of 2 × 10^6^ cells in 200 *μ*L.

MRI exams of SPIO-aPD-L1-Cy5.5 NPs were processed by a 3.0TMR (GE Healthcare, USA) with a mouse coil (RF TECH LIMITED, China). SPIO-aPD-L1-Cy5.5 NPs range 0–0.714 mg/ml Fe concentrations were dissolved in pure water, added to 1.5 ml EP tubes, and then scanned. T2-weighted MRI was performed for each tube using a fast spin-echo (FSE) sequence (slice thickness of 3 mm, TR/TE 2000/74.4 ms, 8 × 8 cm FOV, and 320 × 256 matrix).

For *in vivo* MRI and NIRF, the nude mice were divided into two groups (consisting of 3 mice), including the experimental and control groups. All mice were scanned twice, before injection and 6 h after injection of 0.1 ml NPs into the tail vein, and the dose of Fe was 2 mg/kg. The fluorescent images were photographed by a fluorescence imaging system (Bruker, USA) (Ex630 nm, Em700 nm, Exposure time 1 min, and 12 × 12 cm FOV). During imaging, mice were exposed to a 2% isoflurane and oxygen gas mixture and maintained under anesthesia. Relative signal intensity (RSI) was defined as the ratio of tumor to muscle signal and used to assess NPs targeting ability.

After the imaging, the mice were euthanized, and the tumor was collected. All tissues were fixed with 10% formalin. Subsequently, tumor specimens of the two groups were removed and paraffin-embedded for 4-*μ*m sectioning. Also, sections were stained with Prussian blue to visualize the accumulation of NPs.

### 2.6. Fe Determination

This part uses Du's [27] method. Fe content in the tissues was determined using inductively coupled plasma mass spectrometry (ICP-MS). Briefly, liver, spleen, and tumor tissues were collected at 6 h after injected via tail vein with NPs and washed with deionized water three times. Then, 100 mg tissues were digested with 1 mL of HNO_3_ (70% HNO_3_ for trace metal analysis), and the concentrations of Fe were determined using ICP-MS. The amount of Fe was shown as mg/kg tissue.

### 2.7. Cellular Toxicity Study *In Vivo*

We monitored the *in vivo* toxicity of the NPs by observing the presence of damage within the sections by H&E staining. Blank Balb/c nude mice (aged 4 weeks) were divided into 2 groups (*n* = 3), and SPIO-aPD-L1-Cy5.5 and SPIO-IgG-Cy5.5 were injected. After 24 h, mice were sacrificed, and their hearts, livers, spleens, lungs, and kidneys were harvested. The obtained sections were stained with H&E staining, which helps us see if there is tissue damage.

### 2.8. Statistical Analysis

Each group of data was obtained by repeating three times and expressed as mean ± standard deviation (*x* ± *s*). Independent samples *t*-test was performed using SPSS 23.0 software to compare RSI and Fe concentration between the SPIO-aPD-L1-Cy5.5 group and SPIO-IgG-Cy5.5 group. The statistical significance of the difference was expressed as *p* < 0.05.

## 3. Results

### 3.1. Characterization of the SPIO-aPD-L1-Cy5.5 Nanoprobe

**Figure 1:**
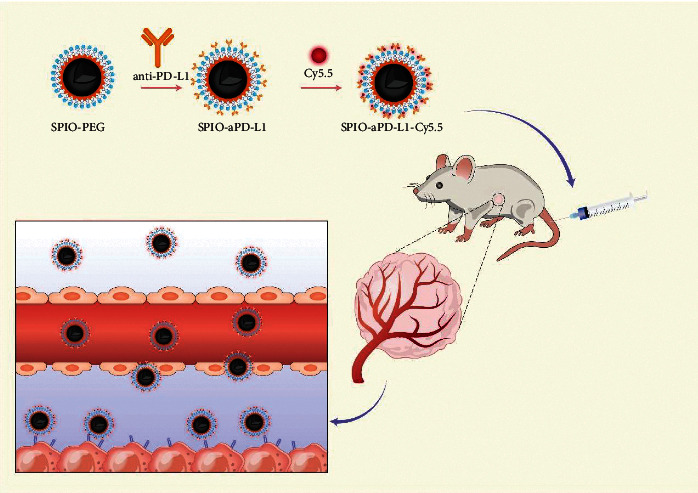
Schematic illustration of in situ imaging and targeted binding mechanism of SPIO-aPD-L1-Cy5.5.

**Figure 2:**
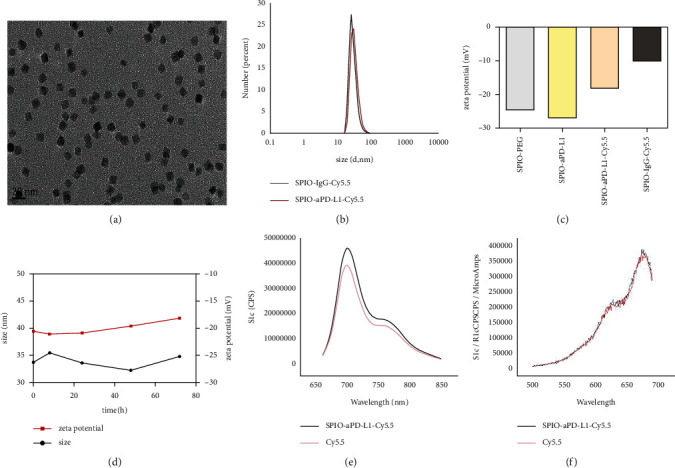
Characterization of SPIO-aPD-L1-Cy5.5. (a) TEM imaging, (b) the mean sizes, (c) the zeta potential, (d) the time-dependent size and zeta potential of SPIO-aPD-L1-Cy5.5, (e) the emission spectra, and (f) the excitation spectra.

SPIO-aPD-L1-Cy5.5 was obtained according to the normal procedures (Figure 1). The SPIO-aPD-L1-Cy5.5 solution was a clear grey liquid with no significant precipitates, suggesting good dispersion after standing for a while. Transmission electron microscopy (TEM) revealed spherical-shaped NPs with a relatively uniform size. The DLS showed that the sizes of SPIO-aPD-L1-Cy5.5 and SPIO-IgG-Cy5.5 were 29.78 nm and 26.70 nm, respectively. As shown in Figure 2, the nanoparticles exhibited a narrow size distribution in water and good dispersion. The zeta potential of SPIO-aPD-L1-Cy5.5 was −27.1 mV and −18.0 mV before and after coupling Cy5.5 since Cy5.5 is a hydrophobic molecule. And the zeta potential of SPIO-IgG-Cy5.5 was −10.26 mV (Figure 2(c)). The obtained NPs exhibit high stability for 5 time points since no significant variations of their particle size and zeta potential were observed (Figure 2(d)).

The results of fluorescence spectra for the NPs coupled with fluorescent Cy5.5 with excitation wavelengths of 670 nm exhibited a maximum emission peak at 698 nm (Figures 2(e) and 2(f)), consistent with the excitation and emission wavelength of fluorescent Cy5.5. The fluorescence spectrum and change of surface zeta potential indicated that Cy5.5 was successfully coupled with the NPs.

### 3.2. Cell Viability

**Figure 3:**
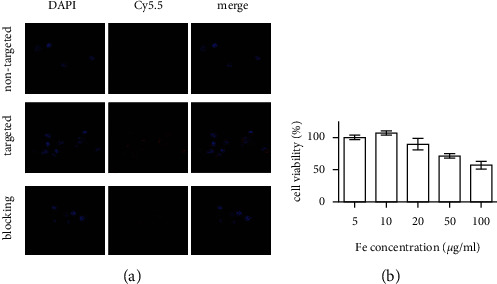
(a) Confocal fluorescence images of 4T1 cells incubated with SPIO-aPD-L1-Cy5.5 (targeted group) and SPIO-IgG-Cy5.5 (nontargeted group). (b) The viability of 4T1 cells incubated with different concentrations of SPIO-aPD-L1-Cy5.5.

We estimated the *in vitro* cytotoxicity of NPs on 4T1 cells through CCK8 assays. Our results showed that the viability of cells is negatively related to the concentration, and their cell viability gradually decreased as the concentration of SPIO-aPD-L1-Cy5.5 was increased. At the highest concentration, 4T1 cells exhibited a significant decrease in cell viability (approximately 50%) (Figure 3). Accordingly, we conclude that SPIO-aPD-L1-Cy5.5 NPs exhibit slight cytotoxicity on 4T1 cells over a given concentration range and were positively correlated with the SPIO-aPD-L1-Cy5.5 concentration.

### 3.3. Cellular Uptake Study *In Vitro*

A confocal laser scanning microscope (CLSM) was used to view the cellular uptake of the SPIO-aPD-L1-Cy5.5.4T1 cells treated with SPIO-aPD-L1-Cy5.5 displayed noticeable red fluorescence enhancement in the membrane. And blocking group demonstrated significantly reduced fluorescence signal. As a control, cells treated with SPIO-IgG-Cy5.5 showed significantly lower red fluorescence signals. And the results of the blocking and control groups indicated that the PD-L1 targeting strategy effectively enhanced the uptake of SPIO-aPD-L1-Cy5.5 by 4T1 cells (Figure 3(a)).

### 3.4. *In Vitro* and *In Vivo* MR Imaging

**Figure 4:**
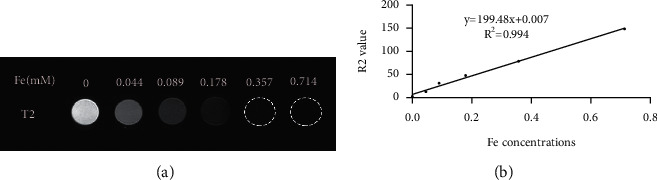
The relaxation properties of SPIO-aPD-L1-Cy5.5 nanoprobes. (a) T2WI images of SPIO-aPD-L1-Cy5.5 in different concentrations. (b) The fitting curves of Fe concentration.

We used T2-weighted MR imaging to determine the T2-weighted relaxivity (R2 value) for the MRI potential of SPIO-aPD-L1-Cy5.5. From Figure 4(a), the T2-weighted MR image darkened with an increase in Fe concentration. To further assess the presence of a linear relationship, a quantitative analysis was performed. SPIO-aPD-L1-Cy5.5 showed a high R2 value which reached 199.48 s^−1^ mM^−1^. These results suggested that SPIO-aPD-L1-Cy5.5 could be applied as a sensitive MRI T2 contrast agent to visualize the *in vivo* drug delivery process.

**Figure 5:**
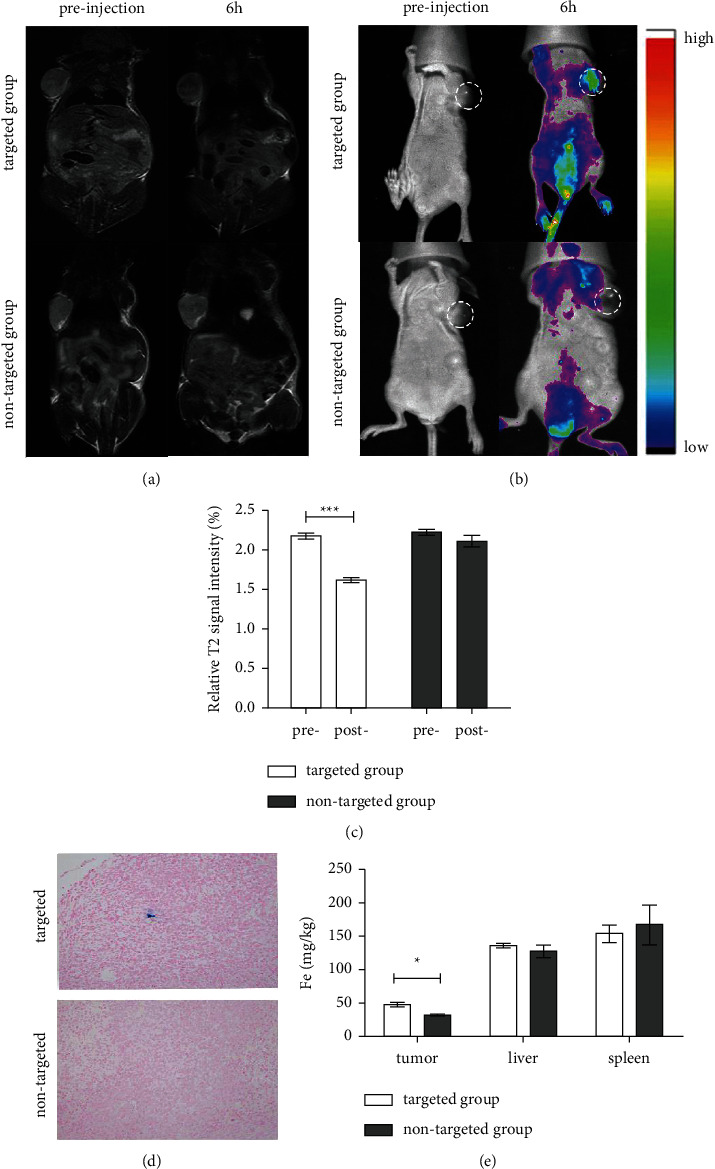
*In vivo* MR T2WI images and fluorescence images of the 4T1-bearing mouse model. (a, b) MR images and fluorescence images of 4T1-bearing mice before and after injection with SPIO-aPD-L1-Cy5.5 (targeted group) and SPIO-IgG-Cy5.5 (nontargeted group). (c) The signal intensity ratio is calculated by dividing the signal intensity of the tumor tissue by the signal intensity of the muscle (reference). (d) Prussian blue staining of tumor tissues. (a) SPIO-aPD-L1-Cy5.5, (b) SPIO-IgG-Cy5.5, and (e) Fe concentrations in the tumor tissues, liver, and spleen after injection of SPIO-aPD-L1-Cy5.5 and SPIO-IgG-Cy5.5 (*n* = 3). ^*∗*^*p* < 0.05 vs. SPIO-IgG-Cy5.5.

Then, we studied the *in vivo* contrast by intravenous injection of SPIO-aPD-L1-Cy5.5 NPs in 4T1-bearing mice. The T2 signals at the tumor sites were darker 6h after injection. In addition, we analyzed the relative signal intensity (RSI) between the two groups (preinjection and postinjection). The results suggested that the preinjection and postinjection RSI in the targeted group was 2.18 and 1.62, respectively, and the difference was statistically significant (*p* < 0.05). Besides, few iron nanoparticles were deposited in the tumor tissue in the targeted group (Figure 5(d)), which could explain the decrease in T2 signal in the SPIO-aPD-L1-Cy5.5 group.

### 3.5. ICP-MS

The distribution of Fe in tissues was validated by ICP-MS. The liver and spleen had the greatest accumulation of Fe (Figure 5(e)); however, there were no differences between the two groups (*p* > 0.05). The Fe levels in the tumor tissue in the SPIO-aPD-L1-Cy5.5 group were significantly higher than in the SPIO-IgG-Cy5.5 group (*p* < 0.05).

### 3.6. *In Vivo* NIRF

In 4T1-bearing nude mice that received SPIO-aPD-L1-Cy5.5, NIRF signals were detected in the whole body. Besides, strong NIRF signals were observed in the tumor region in the targeted group at 6 h (Figure 5(b)), in contrast with the nontargeted group.

### 3.7. H&E Staining

**Figure 6:**
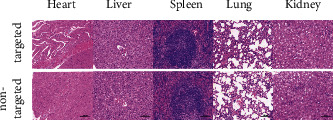
H&E staining of tissue slices of the main organs collected from mice after the injection of SPIO-aPD-L1-Cy5.5 (targeted group) and SPIO-IgG-Cy5.5 (nontargeted group).

Moreover, H&E staining of SPIO-aPD-L1-Cy5.5 NPs showed normal pathological morphology and no histopathological damage response in tissue sections of all organs, compared with the nontargeted group (Figure 6). The cytotoxicity and histological analysis results indicated that SPIO-aPD-L1-Cy5.5 NPs induced no significant toxicity to major organ tissues *in vivo*.

## 4. Discussion

It has been established that PD-L1 is upregulated in TNBC, and its expression is negatively associated with poor patient prognosis and correlates with ICI treatment response rate. Therefore, an accurate means of monitoring PD-L1 expression at the tumor site can prevent PD-1/PD-L1 responders from discontinuing immunotherapy due to erroneous PD-L1 results [28, 29]. However, many limitations surround the assessment of PD-L1 expression. In the present study, a dual-mode molecular probe was synthesized for real-time and dynamic detection of PD-L1 expression, which yielded an excellent predictive performance.

MRI is a well-established method in breast imaging, with various clinical applications, including the noninvasive differentiation between benign and malignant breast lesions, treatment effect evaluation, and the evaluation of high-risk patients. Commonly used contrast agents are broadly divided into T1-positive and T2-negative contrast agents. SPIO is widely acknowledged as a superparamagnetic material that provides a rapid reduction in the intensity of the T2 signal. Liu et al. [30] employed PEG nanoparticles as carriers to couple sLex to SPIO compounds to synthesize a molecule probe, SPIO-PEG-sLex, which could effectively decrease the T2^*∗*^ value. In the present study, the NPs SPIO-aPD-L1-Cy5.5 synthesized was found to have a T2 relaxation rate of 199.48 s^−1^ mM^−1^; importantly, this complex exhibited excellent MR imaging properties *in vivo*.

The surface chemistry and size of SPIO nanoparticles can influence their biodistribution pattern and circulation time in the body. The biodistribution patterns of SPIO nanoparticles have been mostly characterized in the liver and spleen [25, 31]. Studies have shown that to avoid liver and spleen capture and prolong the blood circulation time, the size of nanoparticles should be smaller than 200 nm. On the other hand, the size of nanoparticles should be larger than 10 nm to evade kidney filtration. Accordingly, a range of 10–100 nm is optimal for intravenous injection [32–34]. The size of our synthesized particles is within this range. To further reduce the uptake by the liver and kidneys, our particles were conjugated to polyethylene glycol (PEG) to avoid absorption and increase the blood circulation half-life [35].

The nanoprobe prepared by our group exhibited a relatively uniform and appropriate size. Furthermore, we demonstrated the biocompatibility and molecular imaging of NPs. The study showed that both targeted and nontargeted nanoparticles could effectively reduce the T2^*∗*^ value at the tumor site, especially the targeted nanoparticles. The nanoparticles prepared by our group reached the tumor site via its enhanced permeation and retention (EPR) effects [36, 37]. The enhanced permeability and retention (EPR) effect is an important phenomenon in solid tumors that is related to chaotic angiogenesis and wide vascular endothelial cell gap, which lead to tumor tissues showing considerable extravasation of nanomedicines [38]. Active targeting can be used as a complementary strategy to improve nanomedicine tumor accumulation and retention [39, 40]. Therefore, our strategy of conjugating PD-L1 successfully increased the accumulation of targeted tumors.

In the present study, the T2-negative contrast agent SPIO exhibited more sensitive signal changes than the Gd agent while avoiding the nephrotoxicity associated with the latter. Moreover, the synthesized NPs allow effective, noninvasive, and real-time detection of the expression of PD-L1 in the tumor tissue. However, the relationship between the PD-L1 expression and changes in the MR signals was not quantified, warranting further studies.

## 5. Conclusion

In conclusion, our group designed a dual-modality PD-L1-targeted nanoparticle probe, which exhibited slight cytotoxicity and could specifically bind to the cell surface. Furthermore, we validated its biocompatibility and targeted ability by NIRF/MR imaging *in vivo* and H&E staining. This work provides the basis for future studies on molecular imaging of PD-L1 in cancer patients.

## Data Availability

The data used to support the study are included within the article.

## Abbreviations

Cy5.5: Sulfo-cyanine5.5
DMF: N-dimethylformamide
EDC: 1-Ethyl-3-(3-dimethylaminopropyl) carbodiimide hydrochloride
ICI: Immune checkpoint inhibition therapy
NHS: N-hydroxysuccinimide
NIRF: Near-infrared fluorescence imaging
NPs: Nanoparticles
MR: Magnetic resonance
PBS: Phosphate buffered saline
PCR: Pathologic complete response
PD-1: Programmed cell death protein 1
PD-L1: Programmed cell death-ligand 1
PEG: Polyethylene glycol
SPIO: Superparamagnetic iron oxide
TEM: Transmission electron microscopy
TNBC: Triple-negative breast cancer
TE: Echo time
TR: Repetition time.
